# Supplementary material for: Modeling glioblastoma heterogeneity as a dynamic network of cell states
Source: Mol Syst Biol. 2021 Sep 16;17(9):e10105. doi: 10.15252/msb.202010105 (PMC8444284; doi:10.15252/msb.202010105)
Supplement: Supplementary file 6 — Source Data for Figure 5 [file MSB-17-e10105-s004.zip › Figure5A_sourcedata/GSEA_3071/nosigdb_stateI.GseaPreranked.1622795145664/IVY_CTMVP.html]

Details for gene set IVY\_CTMVP[GSEA]

|  || Dataset | state13071 |
| Phenotype | NoPhenotypeAvailable |
| Upregulated in class | na\_pos |
| GeneSet | IVY\_CTMVP |
| Enrichment Score (ES) | 0.7140372 |
| Normalized Enrichment Score (NES) | 4.0856857 |
| Nominal p-value | 0.0 |
| FDR q-value | 0.0 |
| FWER p-Value | 0.0 |
Table: GSEA Results Summary

  

Fig 1: Enrichment plot: IVY\_CTMVP      
 Profile of the Running ES Score & Positions of GeneSet Members on the Rank Ordered List

  

| PROBE | GENE SYMBOL | GENE\_TITLE | RANK IN GENE LIST | RANK METRIC SCORE | RUNNING ES | CORE ENRICHMENT || 1 | MGP |  |  | 1 | 2.652 | 0.0365 | Yes |
| 2 | COL3A1 |  |  | 3 | 2.646 | 0.0730 | Yes |
| 3 | LUM |  |  | 4 | 2.203 | 0.1035 | Yes |
| 4 | DCN |  |  | 5 | 2.010 | 0.1314 | Yes |
| 5 | COL1A1 |  |  | 6 | 2.008 | 0.1593 | Yes |
| 6 | PDGFRB |  |  | 9 | 1.893 | 0.1850 | Yes |
| 7 | FN1 |  |  | 11 | 1.769 | 0.2092 | Yes |
| 8 | COL6A3 |  |  | 13 | 1.717 | 0.2328 | Yes |
| 9 | PCOLCE |  |  | 15 | 1.696 | 0.2560 | Yes |
| 10 | TGFBI |  |  | 17 | 1.674 | 0.2790 | Yes |
| 11 | BGN |  |  | 24 | 1.568 | 0.2990 | Yes |
| 12 | COL4A1 |  |  | 28 | 1.524 | 0.3193 | Yes |
| 13 | CFH |  |  | 29 | 1.523 | 0.3404 | Yes |
| 14 | MFAP2 |  |  | 44 | 1.395 | 0.3557 | Yes |
| 15 | IGFBP7 |  |  | 45 | 1.387 | 0.3749 | Yes |
| 16 | COL5A2 |  |  | 48 | 1.373 | 0.3934 | Yes |
| 17 | CTHRC1 |  |  | 49 | 1.370 | 0.4124 | Yes |
| 18 | OLFML2A |  |  | 68 | 1.227 | 0.4242 | Yes |
| 19 | COL4A2 |  |  | 73 | 1.203 | 0.4398 | Yes |
| 20 | CD248 |  |  | 79 | 1.190 | 0.4548 | Yes |
| 21 | F2R |  |  | 105 | 1.067 | 0.4623 | Yes |
| 22 | S1PR3 |  |  | 114 | 1.043 | 0.4745 | Yes |
| 23 | SVIL |  |  | 120 | 1.014 | 0.4871 | Yes |
| 24 | OLFML3 |  |  | 125 | 0.971 | 0.4994 | Yes |
| 25 | GPX8 |  |  | 133 | 0.959 | 0.5107 | Yes |
| 26 | KDELR3 |  |  | 136 | 0.951 | 0.5233 | Yes |
| 27 | SNAI2 |  |  | 142 | 0.940 | 0.5349 | Yes |
| 28 | CTGF |  |  | 152 | 0.913 | 0.5449 | Yes |
| 29 | SERPINH1 |  |  | 157 | 0.902 | 0.5563 | Yes |
| 30 | ITGA1 |  |  | 160 | 0.891 | 0.5681 | Yes |
| 31 | PCDH18 |  |  | 171 | 0.870 | 0.5772 | Yes |
| 32 | LAMA4 |  |  | 176 | 0.863 | 0.5881 | Yes |
| 33 | ISLR |  |  | 183 | 0.853 | 0.5981 | Yes |
| 34 | EBF1 |  |  | 221 | 0.783 | 0.5982 | Yes |
| 35 | OLFML2B |  |  | 233 | 0.763 | 0.6056 | Yes |
| 36 | TM4SF1 |  |  | 249 | 0.735 | 0.6115 | Yes |
| 37 | PLAC9 |  |  | 254 | 0.731 | 0.6205 | Yes |
| 38 | PRKG1 |  |  | 261 | 0.726 | 0.6288 | Yes |
| 39 | IGFBP4 |  |  | 262 | 0.725 | 0.6388 | Yes |
| 40 | PRSS23 |  |  | 264 | 0.721 | 0.6486 | Yes |
| 41 | FOXS1 |  |  | 282 | 0.698 | 0.6533 | Yes |
| 42 | UACA |  |  | 296 | 0.679 | 0.6589 | Yes |
| 43 | ACTG2 |  |  | 313 | 0.659 | 0.6634 | Yes |
| 44 | TPM2 |  |  | 320 | 0.655 | 0.6707 | Yes |
| 45 | COL18A1 |  |  | 336 | 0.638 | 0.6752 | Yes |
| 46 | PROCR |  |  | 355 | 0.627 | 0.6787 | Yes |
| 47 | ITGB1 |  |  | 371 | 0.615 | 0.6829 | Yes |
| 48 | FILIP1L |  |  | 387 | 0.602 | 0.6869 | Yes |
| 49 | LAMB1 |  |  | 415 | 0.577 | 0.6870 | Yes |
| 50 | CRIP1 |  |  | 416 | 0.577 | 0.6950 | Yes |
| 51 | MYL9 |  |  | 417 | 0.576 | 0.7030 | Yes |
| 52 | MYO1B |  |  | 441 | 0.556 | 0.7040 | Yes |
| 53 | EDNRA |  |  | 479 | 0.529 | 0.7006 | Yes |
| 54 | ARHGAP18 |  |  | 501 | 0.510 | 0.7015 | Yes |
| 55 | FSTL1 |  |  | 505 | 0.504 | 0.7077 | Yes |
| 56 | ARHGAP29 |  |  | 508 | 0.502 | 0.7140 | Yes |
| 57 | PDLIM1 |  |  | 539 | 0.487 | 0.7121 | No |
| 58 | PDE5A |  |  | 608 | 0.447 | 0.6985 | No |
| 59 | THBS1 |  |  | 613 | 0.446 | 0.7035 | No |
| 60 | LAMC1 |  |  | 640 | 0.439 | 0.7020 | No |
| 61 | PXDN |  |  | 690 | 0.416 | 0.6935 | No |
| 62 | NR2F2 |  |  | 741 | 0.400 | 0.6845 | No |
| 63 | PAPSS2 |  |  | 898 | 0.351 | 0.6439 | No |
| 64 | PDGFD |  |  | 1082 | 0.311 | 0.5949 | No |
| 65 | PHACTR2 |  |  | 1142 | 0.299 | 0.5819 | No |
| 66 | TGFBR2 |  |  | 1410 | 0.251 | 0.5076 | No |
| 67 | RAMP2 |  |  | 1566 | -0.263 | 0.4661 | No |
| 68 | KCNJ8 |  |  | 1601 | -0.268 | 0.4599 | No |
| 69 | APOLD1 |  |  | 1781 | -0.291 | 0.4118 | No |
| 70 | A2M |  |  | 1808 | -0.295 | 0.4083 | No |
| 71 | ETS1 |  |  | 2086 | -0.332 | 0.3322 | No |
| 72 | MCAM |  |  | 2310 | -0.374 | 0.2725 | No |
| 73 | KCNJ2 |  |  | 2528 | -0.422 | 0.2151 | No |
| 74 | GNG11 |  |  | 2554 | -0.427 | 0.2138 | No |
| 75 | PLXDC1 |  |  | 2835 | -0.514 | 0.1394 | No |
| 76 | CCDC102B |  |  | 3221 | -0.807 | 0.0384 | No |
| 77 | INPP4B |  |  | 3263 | -0.871 | 0.0385 | No |
| 78 | STC1 |  |  | 3349 | -1.043 | 0.0283 | No |
| 79 | PDGFB |  |  | 3428 | -1.343 | 0.0242 | No |
Table: GSEA details [plain text format]

  

Fig 2: IVY\_CTMVP: Random ES distribution      
 Gene set null distribution of ES for **IVY\_CTMVP**
